# 2848. Risk factors for recurrence of community-onset urinary tract infections caused by extended spectrum cephalosporin-resistant Enterobacterales

**DOI:** 10.1093/ofid/ofad500.2458

**Published:** 2023-11-27

**Authors:** Helen L Zhang, Reinaldo Perez, Jay Krishnan, Ebbing Lautenbach, Deverick J Anderson

**Affiliations:** Duke University, Durham, North Carolina; Duke University, Durham, North Carolina; Duke University, Durham, North Carolina; University of Pennsylvania, Philadelphia, Pennsylvania; Duke Center for Antimicrobial Stewardship and Infection Prevention, Durham, North Carolina

## Abstract

**Background:**

Extended spectrum cephalosporin-resistant Enterobacterales (ESCR-E) are increasingly implicated in community-onset urinary tract infections (UTIs). In this study, we assessed risk factors for recurrence among patients with community-onset UTI caused by ESCR-E.

**Methods:**

This retrospective cohort study included adult patients evaluated April 2018 – December 2021 in the Duke University Health System with community-onset ESCR-E UTI, defined as (1) ESCR-E in a urine culture obtained in an outpatient clinic, emergency department, or within 48 hours of hospital admission; (2) ≥10 leukocytes per high-power field on urine microscopy or urine dipstick positive for leukocyte esterase; and (3) new antibiotic administration or prescription. ESCR-E UTI recurrence was assessed 14 to 180 days after completion of antibiotic treatment for the index UTI. Patients were right censored at end of follow up period or upon death. Univariate Cox proportional hazards regression was performed to evaluate the relationships between candidate risk factors and time to recurrence.

**Results:**

1428 patients were included; 207 (14.5%) experienced recurrence. In unadjusted analyses, risk factors for recurrent ESCR-E UTI included diabetes mellitus (hazard ratio [HR] = 1.4, 95% confidence interval [CI]: 1.1 to 1.9, p = 0.01), chronic renal insufficiency (HR = 1.6, 95% CI: 1.2 to 2.1, p < 0.001), neurogenic bladder (HR = 2.1, 95% CI: 1.4 to 2.9, p < 0.001), previous UTI diagnosis within one year (HR = 2.6, 95% CI: 1.9 to 3.5, p < 0.001), fluoroquinolone non-susceptibility (HR = 1.6, 95% CI: 1.2 to 2.2, p = 0.004), and trimethoprim-sulfamethoxazole non-susceptibility (HR = 1.3, 95% CI: 1.0 to 1.8, p = 0.045). Patients with ESCR *Klebsiella pneumoniae* UTI had a 53% greater hazard of recurrence compared to patients with ESCR *E. coli* UTI (HR = 1.5, 95% CI: 1.1 to 2.1, p = 0.008).

**Conclusion:**

ESCR-E UTI recurrence was common, and several clinical and microbiologic characteristics were associated with recurrence. Patients with these characteristics should receive particular consideration for aggressive UTI risk factor modification and other non-antibiotic prevention strategies. Future studies should evaluate strategies to reduce the risk of recurrence among patients with ESCR-E UTI.

**Disclosures:**

**All Authors**: No reported disclosures

